# Challenges and Opportunities of Early Brachial Plexus Reconstruction in Polytrauma: Case Report and Review of the Literature

**DOI:** 10.3390/jcm15031300

**Published:** 2026-02-06

**Authors:** Martina Giacalone, Fabrizio Fiumedinisi, Richard Glaab, Regula Marti, Jan A. Plock, Florian S. Frueh

**Affiliations:** 1 Department of Plastic Surgery and Hand Surgery, Kantonsspital Aarau, 5001 Aarau, Switzerland; martina.giacalone@students.uniroma2.eu (M.G.);; 2 Plastic and Reconstructive Surgery, Department of Surgical Sciences, Tor Vergata University of Rome, 00133 Rome, Italy; 3 Department of Orthopaedics and Traumatology, Kantonsspital Aarau, 5001 Aarau, Switzerland; 4 Department of Vascular Surgery, Kantonsspital Aarau, 5001 Aarau, Switzerland

**Keywords:** brachial plexus, brachial plexus injury, nerve grafting, nerve transfer, polytrauma

## Abstract

**Background:** Assessment and treatment of brachial plexus injury in polytrauma patients is often challenging due to concomitant injuries requiring life-saving interventions. Furthermore, the role of immediate nerve exploration in closed postganglionic nerve injuries remains debated. **Case presentation:** We present the case of a 21-year-old male with an infraclavicular brachial plexus injury, floating shoulder and axillary artery rupture following a motorcycle accident. Early multidisciplinary intervention included vascular repair, bone stabilization and brachial plexus exploration. Nerve reconstruction using grafts and transfers led to significant functional recovery, preventing degenerative changes, and facilitating early rehabilitation. **Conclusions:** This case highlights the benefits of early exploration and management of complex brachial plexus injuries in polytrauma patients to improve functional outcomes and quality of life.

## 1. Introduction

Traumatic brachial plexus injuries (BPIs) are commonly associated with long-term upper limb functional disability, chronic pain, and significant socioeconomic burden. These injuries predominantly affect young males involved in high-speed motorcycle accidents, with a reported prevalence ranging from 0.3 to 5% among polytrauma patients [1,2,3,4].

Traumatic BPIs usually result from high-energy mechanisms, including motor vehicle accidents, ballistic trauma, or falls from height, with injury occurring primarily through traction, compression, or direct disruption of the plexus [5].

The complexity of traumatic BPI is further increased when associated with severe skeletal injuries of the shoulder girdle. In particular, the so-called floating shoulder represents a double disruption of the superior shoulder suspensory complex (SSSC). As described by Goss, the presence of two disrupted elements of the SSSC may result in mechanical instability and impaired healing [6]. Within this framework, clavicular fractures play a pivotal role, because disruption of the clavicular strut or coracoclavicular ligaments compromises the integrity of the SSSC and contributes to shoulder instability [6]. According to the Goss classification, such injury patterns have relevant implications for surgical decision-making and prognosis [7]. The coexistence of proximal humeral fractures may further aggravate this instability by altering load transmission and scapulohumeral alignment, thereby negatively influencing functional recovery and delaying rehabilitation [8].

The loss of bony and ligamentous constraints allows abnormal translation of the scapulohumeral complex, increasing the risk of concomitant infraclavicular brachial plexus and axillary artery injuries [9]. While the incidence of this association remains low and is mainly reported in case reports, its presence has major implications for early diagnosis, surgical timing, and multidisciplinary management.

In these polytrauma scenarios, early recognition and assessment of BPIs are often challenging, as life-threatening injuries take priority and often delay referral to specialized centers. Early evaluation of a BPI by an experienced brachial plexus surgeon is crucial for an effective treatment, as timely reconstruction significantly impacts the outcome of these devastating injuries. For closed in-continuity injuries, non-surgical conservative treatment is recommended to wait for spontaneous recovery [10]. In contrast, high-grade BPI, i.e., severe axonotmesis or neurotmesis, or open BPI should be explored and reconstructed as soon as possible. While the benefits of early exploration remain a subject of debate, delays in surgery are known to progressively diminish reconstructive outcomes due to the declining regenerative capacity of motor nerve axons, loss of motor end-plates from denervation, and progressive muscle atrophy [11,12]. Surgical delay should therefore be avoided when spontaneous recovery appears unlikely.

This case report describes a rare association of infraclavicular brachial plexus injury, floating shoulder, and axillary artery rupture in a polytrauma setting. It aims to provide insight into the feasibility of early multidisciplinary management, including brachial plexus exploration and reconstruction.

## 2. Case Presentation

A 21-year-old male was admitted to the emergency department following a high-velocity motorcycle accident (day 0). On admission, the patient was conscious with patent airways but complained of severe thoracic and abdominal pain. Clinical examination revealed complete paralysis of the right upper limb and absence of radial and ulnar artery pulses, raising suspicion of severe neurovascular injury.

### 2.1. Initial Assessment and Emergency Surgical Management (Day 0)

**Figure 1:**
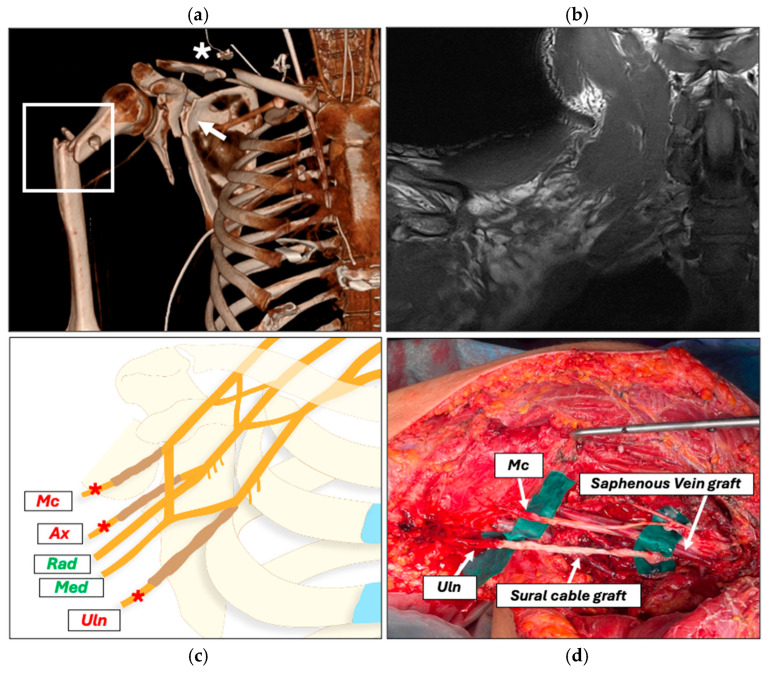
Preoperative assessment and interdisciplinary reconstruction. (**a**) CT showing a floating shoulder injury with fracture of the right clavicle (asterisk), scapular neck (arrowhead) and proximal humerus (rectangle). (**b**) MRI showing a right infraclavicular hematoma not allowing the assessment of the infraclavicular brachial plexus. (**c**) Schematic illustration of injured nerves (red asterisks) (Mc: musculocutaneous; Ax: axillary; Uln: ulnar) and intact nerves shown in green (Rad: radial; Med: median). (**d**) Intraoperative picture illustrating the reconstruction of the musculocutaneous and ulnar nerve and reverse saphenous vein graft for axillary artery repair.

Ultrasound assessment demonstrated a right-sided hemopneumothorax and diffuse intra-abdominal bleeding. Due to hemodynamic instability, immediate chest tube placement was performed, followed by urgent transfer to the operating room for damage control surgery. After initial stabilization, a whole-body trauma computed tomography scan was obtained, demonstrating a floating shoulder injury with fractures of the right clavicle, scapular neck, and proximal humerus, associated with multiple rib fractures and rupture of the right axillary artery (Figure 1a).

Following CT confirmation of major vascular injury, the patient was transferred to the operating room for emergency multidisciplinary surgery. Revascularization of the right upper limb was achieved using a temporary polytetrafluoroethylene (PTFE) bypass from the right common carotid artery to the radial artery. Concomitantly, forearm fasciotomies were performed, and the proximal humeral fracture was managed with closed reduction and external fixation.

### 2.2. Neurological Reassessment

Postoperative clinical evaluation of the right upper limb was initially limited by prolonged intubation. At postoperative day (POD) 10, only minimal extrinsic flexion of the index and middle fingers could be observed, with an otherwise completely paralyzed extremity. These findings, in line with the high-energy trauma and the associated injuries, raised suspicion for a high-grade brachial plexus injury.

Magnetic resonance imaging of the cervical spine and brachial plexus ruled out cervical root avulsions but was inconclusive for postganglionic nerve injury due to the presence of an extensive retroclavicular hematoma (Figure 1b). Electromyography and nerve conduction studies were not performed in the acute phase because of their limited diagnostic interpretability (i.e., distinguishing demyelinating from axonal injuries) and limited usefulness in guiding early surgical decision-making.

### 2.3. Definitive Surgical Strategy

Once the patient was stabilized, early definitive interdisciplinary exploration was planned. At POD 11, the axillary artery was reconstructed using a reversed saphenous vein graft, replacing the temporary PTFE bypass. Open reduction and plate osteosynthesis of the humerus and clavicle were performed. Brachial plexus exploration confirmed neurotmesis at the infraclavicular level, including 7–8 cm defects of the ulnar and musculocutaneous nerve and distal avulsion of the axillary nerve, not accessible through an anterior approach. The median and radial nerve were found to be in continuity. Medial antebrachial cutaneous and sural nerve cable grafts were used to reconstruct the biceps branch of the musculocutaneous nerve and the ulnar nerve, respectively (Figure 1c,d).

**Figure 2:**
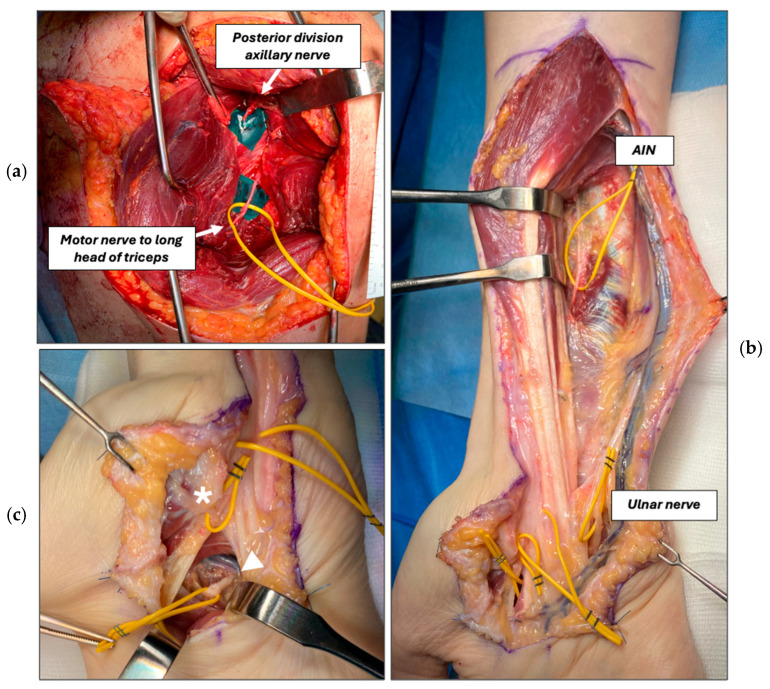
Nerve transfer reconstruction. (**a**) Exploration and dissection of the posterior division of the axillary nerve and motor nerve branch (yellow loop) to the long head of the triceps for Somsak nerve transfer. (**b**) Dissection of the anterior interosseous nerve (AIN) and ulnar nerve for AIN-to-deep ulnar nerve transfer. (**c**) Dissection of the opponens pollicis motor branch (asterisk) and terminal division of the deep branch of the ulnar nerve (arrowhead) for Bertelli nerve transfer.

At POD 16, a triceps-to-axillary nerve transfer (Somsak procedure) [13] was done to reinnervate the deltoid (Figure 2a). For this nerve transfer, a dorsal approach was used, which also allowed to plate the scapula.

Postoperatively, the affected limb was immobilized in a sling for three weeks and shoulder mobilization was avoided. From week 3–4, passive and active-assisted shoulder mobilization was performed within a limited range of motion. Passive mobilization of the hand and wrist was progressively allowed, and assisted elbow movements were initiated. The patient remained hospitalized for 50 days and was subsequently transferred to a trauma rehabilitation center, where he underwent an intensive inpatient rehabilitation program for six months. During this period, the patient underwent occupational therapy three times per week. Therapy included passive, active-assisted, and active mobilization of the hand. Manual and ultrasound-assisted scar treatment was also performed. Sensory retraining and graded motor imagery were incorporated into the rehabilitation. In addition, neuromuscular electrical stimulation was applied to denervated muscle groups. In parallel, the patient received structured psychological support throughout the inpatient rehabilitation period to address emotional distress, pain coping strategies, and adherence to the long-term rehabilitation program. Pain management initially included pregabalin and opioids, with a gradual tapering of the latter until complete discontinuation. After discharge from the rehabilitation center, the patient—who remained compliant throughout the treatment course—continued outpatient rehabilitation three times per week.

To prevent intrinsic muscle loss in high ulnar nerve injury, a transfer of the opponens pollicis thenar branch to the deep branch of the ulnar nerve (DBUN) (Bertelli transfer) [14] and a transfer of the anterior interosseous nerve (AIN) to the deep ulnar nerve branch at the forearm were added 5 months after the initial injury (Figure 2b,c). Postoperative immobilization was maintained for 10 days. Rehabilitation included early-phase electrical stimulation of the reinnervated muscles to prevent muscle atrophy and facilitate neuromuscular activation. This was progressively combined with motor relearning exercises initially based on donor nerve activation, in which contraction of the reinnervated muscle was elicited through voluntary activation and mental imagery of the donor muscle. Subsequently, cortical re-education progressed toward exercises aimed at activating the reinnervated muscle independently from the donor nerve. With progressive functional recovery, rehabilitation advanced to task-specific training and bimanual exercises, integrating both upper limbs, with a focus on activities of daily living and functional use of the reinnervated muscles [15,16].

**Figure 3:**
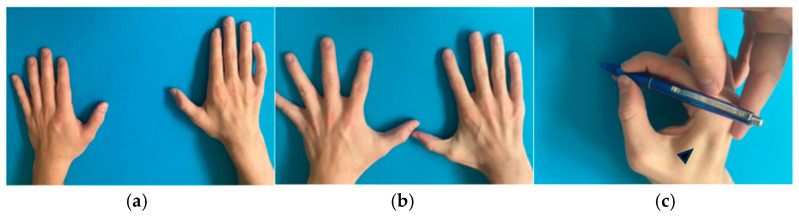
Twelve-month follow-up after intrinsic nerve transfer. (**a**) Dorsal view of injured right and healthy left hand in neutral position with minimal residual clawing (**b**) Finger abduction (**c**) Right hand re-innervated first dorsal interosseous muscle (arrowhead).

At 6 months follow-up, we observed a progressive recovery of shoulder abduction (M4) and elbow flexion (M4), according to the British Medical Research Council (BMRC) scale (Table 1) [17]. At 18 months follow-up, slow but progressive improvement in intrinsic hand muscle function was noted with restoration of 55% of key pinch strength compared to the contralateral healthy side, good restoration of thumb adduction, and weak finger abduction/adduction movements (Figure 3 and Videos S1 and S2). The patient was able to return to his previous professional and recreational activities. Written informed consent was obtained from the patient for publication of clinical data and images.

## 3. Discussion

Postganglionic BPIs in polytrauma patients are often characterized by varying degrees of severity and can be complicated by additional vascular injuries and fractures. Hence, time-consuming nerve exploration and reconstruction may be influenced by the need to prioritize life-threatening injuries [18]. While immediate exploration is indicated for penetrating injuries, the optimal approach to closed injuries remains controversial [19]. Monitoring for spontaneous recovery has been traditionally recommended, yielding good functional outcomes and avoiding unnecessary procedures in neurapraxia and low-grade axonotmesis [12]. However, when high-grade axonotmesis or neurotmesis is suspected, early repair is the most effective option [19].

### 3.1. Challenges and Advantages of Early Exploration

The benefits and limitations of early BPI exploration have been discussed previously, but there is no consensus for this complex problem in patients suffering from polytrauma [10].

We herein presented a case of a young man who sustained a severe postganglionic BPI as part of a polytrauma. Early referral to our unit allowed for a prompt clinical and MRI evaluation, where we suspected a high-grade postganglionic injury. Early diagnosis and assessment of BPI in the context of polytrauma can be difficult due to multiple reasons. First, life-threatening injuries requiring immediate care often lead to delayed referrals. Although evaluation of BPIs is desirable as soon as allowed by a patient’s conditions, there is a trend towards delayed referrals of severely injured patients, leading to suboptimal outcomes of BPI reconstruction [20]. Second, vascular injuries might complicate imaging-based evaluation of the brachial plexus due to fluid extravasation [21]. In line with this, MR imaging, performed in our case, allowed for exclusion of cervical root avulsions but could not adequately depict the infraclavicular plexus due to the presence of a diffuse hematoma. Finally, electrophysiological studies, even though routinely performed in the early posttraumatic period, are not helpful for the planning of early BPI exploration [9]. In our case, based on the clinical suspicion of a high-grade nerve injury, early interdisciplinary exploration was performed with the goal of one-staged arterial, bone and nerve reconstruction.

From a technical perspective, early BPI exploration should only be planned by experienced brachial plexus surgeons, since it requires demanding intraoperative decision-making, mastering multiple reconstructive strategies. For instance, evaluating in-continuity nerve injuries is challenging at this early stage, as persistent nerve conduction block and the absence of neuroma formation make the identification of higher-grade traction injuries difficult [22]. Despite these challenges, early BPI exploration comes along with important advantages. It allows for direct visualization of the nerve injuries in “fresh” tissues, preventing time-dependent degenerative effects on nerve and muscle tissues, which negatively affect reconstructive outcomes [23]. In addition, early exploration also facilitates nerve stump dissection and assessment in a physiological wound bed free of scars when compared to delayed surgery. From a biological point of view, the injured nerves are less retracted without intraneural fibrosis and do not have to be released from the perineural scar, which is commonly found in delayed exploration. These factors should lead to more physiological nerve revascularization, reduced gap lengths and, ultimately, to superior motor and sensory outcomes [10]. Finally, early exploration and reconstruction of brachial plexus injuries in polytrauma patients may also contribute to improved quality of life by facilitating effective rehabilitation and promoting earlier return to daily activities. Pain frequently limits rehabilitation, and timely reconstructive surgery may reduce central exposure to posttraumatic neuropathic pain, possibly leading to lower rates and intensity of pain syndromes [24,25,26]. In the present case, a sequence of nerve grafts and transfers were used to address the functional loss. While nerve grafting remains the standard approach for the reconstruction of postganglionic brachial plexus injuries, nerve transfers have demonstrated comparable outcomes, providing several advantages with expansion of the reconstructive armamentarium [27]. The musculocutaneous and ulnar nerves were reconstructed using nerve grafting, while a Somsak nerve transfer was performed for deltoid reinnervation. Of note, after proximal ulnar nerve cable graft reconstructions, restoration of intrinsic hand function cannot be expected in adults [28]. Consequently, we addressed this problem with additional distal nerve transfers, i.e., the Bertelli and AIN to deep motor branch of ulnar nerve transfers, which have shown promising outcomes in the literature [23]. One year following reconstruction, the reconstructive strategy resulted in restoration of elbow flexion, shoulder abduction, satisfactory grip, pinch strength, and thumb adduction, as well as weak finger abduction and adduction.

### 3.2. The Literature in Context

The combination of floating shoulder and brachial plexus injury is exceedingly rare and the available literature appears to be limited to isolated case reports, underscoring the rarity of this complex trauma pattern (Table 2). Among the published studies, the only case closely comparable to our report is the one by Chen et al., describing a floating shoulder with concomitant axillary artery rupture and acute infraclavicular brachial plexus injury [9]. In their report, management focused on urgent vascular repair and skeletal stabilization. Neurological recovery was described qualitatively and was limited by incomplete patient follow-up.

Venkatramani et al. reported a floating shoulder injury complicated by a delayed onset infraclavicular brachial plexus palsy secondary to mechanical compression from an unhealed clavicular fracture [29]. In that case, neurological deficits developed progressively and were attributed to local compression rather than to an acute traction mechanism. Surgical management consisted of open reduction and internal fixation of the clavicle combined with brachial plexus decompression and neurolysis, performed two months after injury, resulting in good functional recovery. Unlike the delayed compression neuropathy described by Venkatramani et al., our case was characterized by an acute traction-related brachial plexus injury requiring early multidisciplinary management. Vascular repair and stabilization of the shoulder girdle were prioritized and performed in conjunction with early exploration of the brachial plexus, aiming to prevent further neural damage and promptly manage nerve injuries. This comparison highlights two distinct pathophysiological entities within the spectrum of floating shoulder injuries, precluding a meaningful comparison of functional outcome and upper limb function.

Atan et al. reported a “smashed-up shoulder” injury characterized by complex fractures of the clavicle, scapula, and proximal humerus associated with a complete brachial plexus injury, without vascular compromise [30]. Similar to the present case, the mechanism involved high-energy trauma resulting in global shoulder girdle disruption and severe traction forces on the brachial plexus. However, unlike our case, no brachial plexus injury treatment was undertaken and management focused primarily on skeletal stabilization and supportive care. The authors reported no signs of neurological recovery at follow-up, reinforcing the importance of early assessment of brachial plexus injury in this trauma setting to improve long-term upper limb function and minimize functional disability.

In the report by Heng et al., only one of the two described cases involved a true post-ganglionic brachial plexus injury (C5–C8) associated with a high-energy shoulder girdle disruption and concomitant axillary artery injury [31]. Management in this case consisted of early multidisciplinary intervention, including vascular repair, stabilization of the shoulder girdle, and surgical exploration of the brachial plexus. However, detailed neurological and functional outcomes were not systematically reported, limiting comparison in terms of upper limb recovery.

Compared with previously reported cases, the present case is characterized by a favorable and clinically meaningful functional recovery. Importantly, the number of published reports on floating shoulder injuries associated with BPI is limited. In most of these cases, neurological injuries are either not systematically addressed or are managed conservatively, and functional outcomes are frequently underreported. This heterogeneity in reporting substantially limits meaningful comparison across studies and hampers the ability to draw conclusions regarding the optimal timing and strategy for brachial plexus management in this complex trauma setting. However, our case report adds important clinical insight by describing a comprehensive, multidisciplinary approach to the management of combined skeletal, vascular, and peripheral nerve injuries. The integration of early brachial plexus reconstruction within a staged trauma care pathway contributed to a better functional recovery compared with other cases. Collectively, this case highlights the role of early, targeted nerve reconstruction in selected polytrauma patients and underscores the need for a more detailed and standardized reporting of neurological outcomes in future studies.

### 3.3. Limitations and Future Perspectives

This report is limited by its single-case design, which restricts generalizability. Baseline standardized functional scores were not available due to the emergency context, and outcome assessment relied on clinical evaluation and strength grading. Larger series are required to confirm the potential benefits of early brachial plexus exploration in polytrauma patients.

## 4. Conclusions

This article discussed the challenges and opportunities of early exploration and reconstruction of BPIs in a polytrauma case. The reported case suggests that early multidisciplinary evaluation and nerve reconstruction can enhance functional recovery, mitigating degenerative changes and facilitating early rehabilitation. Our experience supports the feasibility of early brachial plexus reconstruction when performed in specialized centers. Considering the technical and functional advantages, early surgical exploration and reconstruction may be considered to enhance outcomes and improve patients’ quality of life, particularly when dealing with high-grade BPIs in polytrauma patients. Integrating proximal nerve grafts and distal nerve transfers allows for individually tailored reconstructions, preventing devastating sequelae such as complete loss of intrinsic hand function.

## Figures and Tables

**Table 1:** British Medical Research Council (BMRC) scale for muscle strength.

**M0**	No muscle contraction
**M1**	Muscle contraction without movement
**M2**	Muscle contraction with movement excluding gravity
**M3**	Active movement against gravity
**M4**	Active movement against gravity and resistance
**M5**	Normal muscle strength

**Table 2:** Reported cases of floating shoulder and brachial plexus injury with or without axillary artery rupture.

	Patient	Injury Type	Floating Shoulder	Axillary Artery Injury	BPI	Nerve Surgery, (Timing)	Neurological Outcome
Chen et al. (2018) [9]	34 y/o, W	Closed injury (motorcycle accident)	Clavicular mid-third, scapula and proximal humerus fractures	Yes	Brachial plexus contusion	None	Satisfactory recovery but limited follow-up
Venkatramani et al. (2020) [29]	65 y/o, M	Closed injury (motorcycle accident)	Clavicular mid-shaft, surgical neck and body scapular fractures	No	Medial and posterior cord compression from non-union of clavicular fracture	BP neurolysis (2 months)	(M4) for deltoid, biceps, triceps, all wrist and digital extensors, all wrist flexors; Grip strength 10 kg, pinch strength 5 kg; Paresthesia resolved; DASH score 15
Atan et al. (2021) [30]	31 y/o, M	Closed injury (motorcycle accident)	Scapula body, clavicular lateral third, and proximal humerus fractures	No	Complete brachial plexus injury	None	No signs of recovery for BPI
Heng K. (2016) [31]	25 y/o, M	Closed injury (motorcycle accident)	Scapula body, distal clavicle and humeral fractures	Yes	Postganglionic C5–C8 injury	BP repair (early)	Not reported

Abbreviations: BPI (brachial plexus injury); DASH (Disabilities of the Arm, Shoulder, and Hand).

## Data Availability

The original contributions presented in this study are included in the article/Supplementary Material. Further inquiries can be directed to the corresponding author.

## Supplementary Materials

The following supporting information can be downloaded at: https://www.mdpi.com/article/10.3390/jcm15031300/s1, Video S1: Postoperative follow-up evaluation at 18 months from brachial plexus reconstruction; Video S2: Postoperative follow-up 20 months after transfer of the opponens pollicis thenar branch to the terminal division of the deep branch of the ulnar nerve and anterior interosseous nerve to the deep ulnar nerve branch.

## Abbreviations

The following abbreviations are used in this manuscript:

BPI	Brachial plexus injury
PTFE	Polytetrafluorethylene
POD	Postoperative day
DBUN	Deep branch of the ulnar nerve
AIN	Anterior interosseous nerve
MRC	Medical Research Council

## References

[1] Faglioni W., Siqueira M.G., Martins R.S., Heise C.O., Foroni L. (2014). The epidemiology of adult traumatic brachial plexus lesions in a large metropolis. Acta Neurochir..

[2] Kaiser R., Mencl L., Haninec P. (2014). Injuries associated with serious brachial plexus involvement in polytrauma among patients requiring surgical repair. Injury.

[3] Zaidman M., Novak C.B., Midha R., Dengler J. (2024). Epidemiology of peripheral nerve and brachial plexus injuries in a trauma population. Can. J. Surg..

[4] Midha R. (1997). Epidemiology of brachial plexus injuries in a multitrauma population. Neurosurgery.

[5] Kim D.H., Murovic J.A., Tiel R.L., Kline D.G. (2004). Mechanisms of injury in operative brachial plexus lesions. Neurosurg. Focus.

[6] Goss T.P. (1993). Double disruptions of the superior shoulder suspensory complex. J. Orthop. Trauma.

[7] van Noort A., van der Werken C. (2006). The floating shoulder. Injury.

[8] Kumar V.P., Satku K. (1993). Fractures of clavicle and scapular neck. J. Bone Jt. Surg. Br. Vol..

[9] Chen Y.C., Lian Z., Lin Y.N., Wang X.J., Yao G.F. (2018). Injury to the axillary artery and brachial plexus caused by a closed floating shoulder injury: A case report. World J. Clin. Cases.

[10] Pondaag W., van Driest F.Y., Groen J.L., Malessy M.J.A. (2019). Early nerve repair in traumatic brachial plexus injuries in adults: Treatment algorithm and first experiences. J. Neurosurg..

[11] Fu S.Y., Gordon T. (1995). Contributing factors to poor functional recovery after delayed nerve repair: Prolonged axotomy. J. Neurosci..

[12] Martin E., Senders J.T., DiRisio A.C., Smith T.R., Broekman M.L.D. (2018). Timing of surgery in traumatic brachial plexus injury: A systematic review. J. Neurosurg..

[13] Rinker B. (2015). Nerve transfers in the upper extremity: A practical user’s guide. Ann. Plast. Surg..

[14] Bertelli J.A. (2024). Reconstructing pinch strength after ulnar nerve injury by transferring the opponens pollicis motor branch. Plast. Reconstr. Surg..

[15] Sturma A., Hruby L.A., Farina D., Aszmann O.C. (2019). Structured motor rehabilitation after selective nerve transfers. J. Vis. Exp..

[16] Novak C.B. (2008). Rehabilitation following motor nerve transfers. Hand Clin..

[17] Medical Research Council (1976). Aids to examination of the peripheral nervous system.

[18] Lam W.L., Fufa D., Chang N.J., Chuang D.C. (2015). Management of infraclavicular (Chuang Level IV) brachial plexus injuries: A single surgeon experience with 75 cases. J. Hand Surg. Eur. Vol..

[19] Burge P., Rushworth G., Watson N. (1985). Patterns of injury to the terminal branches of the brachial plexus. The place for early exploration. J. Bone Jt. Surg. Br..

[20] Morris B.E., Teven C.M., Noland S.S. (2022). Early referral in brachial plexus injury: An ethical obligation. Plast. Reconstr. Surg. Glob. Open.

[21] Szaro P., Geijer M., Ciszek B., McGrath A. (2022). Magnetic resonance imaging of the brachial plexus. Part 2: Traumatic injuries. Eur. J. Radiol. Open.

[22] Frueh F.S., Labèr R., Schiller A., Guidi M., Besmens I.S., Calcagni M., Giovanoli P. (2021). Die intraoperative Faszikel-topografische Elektromyografie in der peripheren Nervenchirurgie—Übersichtsarbeit und Fallbeispiele [The use of intraoperative fascicle-topographic electromyography in peripheral nerve surgery: Review of the literature and clinical experience]. Handchir. Mikrochir. Plast. Chir..

[23] Jivan S., Kumar N., Wiberg M., Kay S. (2009). The influence of pre-surgical delay on functional outcome after reconstruction of brachial plexus injuries. J. Plast. Reconstr. Aesthet. Surg..

[24] Birch R. (2015). Timing of surgical reconstruction for closed traumatic injury to the supraclavicular brachial plexus. J. Hand Surg. Eur. Vol..

[25] Kato N., Htut M., Taggart M., Carlstedt T., Birch R. (2006). The effects of operative delay on the relief of neuropathic pain after injury to the brachial plexus: A review of 148 cases. J. Bone Jt. Surg. Br..

[26] Rasulić L., Savić A., Živković B., Vitošević F., Mićović M., Baščarević V., Puzović V., Novaković N., Lepić M., Samardžić M. (2017). Outcome after brachial plexus injury surgery and impact on quality of life. Acta Neurochir..

[27] Garg R., Merrell G.A., Hillstrom H.J., Wolfe S.W. (2011). Comparison of nerve transfers and nerve grafting for traumatic upper plexus palsy: A systematic review and analysis. J. Bone Jt. Surg. Am..

[28] George S.C., Burahee A.S., Sanders A.D., Power D.M. (2022). Outcomes of anterior interosseous nerve transfer to restore intrinsic muscle function after high ulnar nerve injury. J. Plast. Reconstr. Aesthet. Surg..

[29] Venkatramani H., Bhardwaj P., Raja Sabapathy S., Bandari G., Zhang D., Dheenadhayalan J. (2020). Floating shoulder injury resulting in delayed onset of infraclavicular brachial plexus palsy. J. Hand Surg. Asian-Pac. Vol..

[30] Atan A.A., Ab Rahman Z., Zayzan K.R., Bahaudin N., Ahmad A.R. (2021). The smashed-up shoulder: A case of complex ipsilateral scapula, clavicle and proximal humerus fractures with complete brachial plexus injury. Cureus.

[31] Heng K. (2016). “Floating shoulder” injuries. Int. J. Emerg. Med..

